# A low power, highly efficient, linear, enhanced wideband Class-J mode power amplifier for 5G applications

**DOI:** 10.1038/s41598-022-12235-z

**Published:** 2022-05-16

**Authors:** Nagisetty Sridhar, C. Senthilpari, R. Mardeni, Wong Hin Yong, T. Nandhakumar

**Affiliations:** 1 Faculty of Engineering, Multimedia University (MMU), Cyberjaya, Malaysia; 2 Nottingham University (Malaysia Campus), Semenyih, Malaysia

**Keywords:** Electrical and electronic engineering, Electronic devices

## Abstract

In wireless communication networks, the necessity for high-speed data rates has increased in emerging 5G application areas. The Power Amplifier (PA) topologies reported to date achieved desired Power Added Efficiency (PAE) and linearity. However, these harmonically tuned switching PAs are less appealing for broadband applications as they are restricted to narrow bandwidth (BW). Therefore, to meet the 5G requirements, the challenge of designing a PA with improved efficiency and linearity for a dynamic range of BW becomes critical for PA designers. Recently developed Class-J PA topology can obtain good efficiency while maintaining linearity for wide BW applications. This research work presents a methodology to design a 5 GHz Class-J mode PA topology using Silterra 0.13 μm CMOS technology. This research's main objectives are to determine the R_opt_ of the transistor and design a proper Output Matching Network (OMN) for obtaining Class-J PA operation to make it suitable for 5G wireless applications. The simulation results represent that the designed Class-J PA provides 27 dBm of maximum power output with a maximum power gain of 13.7 dB and the small-signal gain of 17 dB for a BW of around 500 MHz with a 5 V power supply into a 50Ω load.

## Introduction

For the past 40 years, each decade has demanded new technology in the wireless communication industry since introducing the first generation (1G) modern mobile phone systems to fifth-generation (5G) wireless Networks. The emerging 5G technology will enable many new applications commonly termed "5G triangle"^1^. To achieve the requirements of 5G, the research has been started with various techniques like Millimeter waves, Small base stations, Massive MIMO, Beamforming, Full duplexing to implement base stations that will support the “5G triangle” applications^2^.

The essential fact to be considered here is that the power consumed by the mobile base stations (BST) and wireless devices has to be limited to reduce the energy usage for the overall 5G system compared to the existing 4G networks^3^. In addition to this, with a steady increase in the number of subscribers and new application areas that demand higher data rates, there is a need to enhance BW for 5G applications.

It is familiar that the PA is an important block in all RF transmitters as its PAE decides the energy consumed by the overall wireless 5G communication system. Therefore, the emerging 5G cellular network requires advancement in PA architectures^4^ to improve efficiency without sacrificing linearity over a wide dynamic BW. This research aims to analyze, design, and implement a PA that satisfies 5G wireless communication networks.

The main objective of this work is to design a low power, highly efficient, linear, and enhanced broadband integrated PA with a proper OMN that suits 5G Applications. The existing PA structures reported to date^5–11^ demonstrated their potential to achieve promising PAE and linearity results. But these techniques will not benefit the enhanced mobile broadband (EMBB) 5G applications due to their narrow BW nature of harmonic terminations. Designing a PA structure that can achieve high efficiency over a wide BW and linearity is essential. The literature review reveals that out of various modes of PAs, the Class- J PA introduced and developed by S.C.Cripps in Ref.^12^ can provide the BW enhancement required for near future 5G wireless communication applications. This research mainly deals with a Class- J mode PA’s analysis and its design with a proper OMN to obtain the expected performance parameters such as low power consumption, high efficiency without sacrificing linearity, and enhanced wide BW.

In this work, initially, instead of using load-pull simulations, the value of Ropt is calculated using extensive waveform engineering to obtain the fundamental and second harmonic optimum impedances needed for Class-J operation, and then a π**-**type OMN with lumped lossless components is designed to match these optimum impedances with 50Ω load impedance for achieving Class-J mode operation at 5G frequency. The lumped element π**-**matching network was investigated in terms of its BW.

The Class-J PA simulations were performed in the Mentor Graphics EDA tool, and this paper's structure is described as follows. A brief review of the performance parameters achieved by the PAs reported in the literature and the theory of Class-J PA are discussed in second section. The selection of a proper biasing network and the calculation of R_opt_ that is required to find out the optimum impedances to be given to the transistor for obtaining Class-J mode operation is explained in third section. A lumped element-based π**-**type OMN design for matching 50 Ω output load resistance with the Class-J optimum load impedances is presented in fourth section. The simulation results of the lumped-element-based schematic Class-J PA circuit and its active element-based layout design were discussed in fifth section. The conclusion of this research work is briefed in last section.

## Brief review of switching mode PAs and theory of Class-J PA

Many PA structures have been reported with different techniques to improve efficiency and linear amplification. Out of those, the design of a single-stage CMOS-based Class-E PA with cascade topology that operates at 2.4 GHz center frequency for wireless applications is presented in Ref.^5^. Although Class-E PA has circuit simplicity and provides excellent PAE, it has poor linearity due to its switching operation. A 0.13 µm RF CMOS technology-based two-stage PA designed using MOSFET only bias circuits for 2.4 GHz WLAN applications presented in Ref.^6^ achieves reduced power consumption and improved efficiency but still, it exhibits poor linearity. A CMOS-based 24 GHz PA is proposed in Ref.^7^ uses a feedforward canceling path that reduces PA's third-order intermodulation distortion (IMD3). However, there is a small reduction in gain due to the auxiliary path. Various efficiency enhancement and linearization improvement techniques of PAs of mobile base stations, requirements, and recent trends of 5G technology is presented in Ref.^8^. Most of those PAs, such as Class-E, F, etc., implemented for achieving high-efficiency amplification are switching in nature, which may not be suitable for emerging 5G applications such as enhanced mobile broadband (EMBB) due to their narrowband nature and the circuit complexity. Later on, Doherty PA^9,10^ and alternate approaches like stacked and multi-gate cell PAs^11^ are reported with limitations such as narrow BW and circuit complexity, respectively. To provide a solution for such drawbacks, a newly developed Class-J mode PA by Cripps in Ref.^12^ has proved its potentiality in achieving high efficiency and wide BW without sacrificing linearity. A design methodology for highly efficient, linear, and broadband Class-J PA mode is demonstrated in Ref.^13^. The design of 0.5 W GaN-based integrated Class-J PA is presented in Ref.^14^, which takes OMN element’s losses into account for realizing an on-chip output matching, but due to the limitation of device technology and the low-Q on-chip matching network losses, its efficiency and output power are less in comparison with discrete PAs. An integrated CMOS Class-J PA is presented in Ref.^15^, which takes the effect of threshold voltage into account to analyze 2^nd^ harmonic losses for deriving modified design equations. However, the staked FET must be used for implementation because of CMOS PA’s low breakdown voltage. A 28 GHz integrated, highly efficient, and wideband dual-stage Class -J PA is presented in Ref.^16^, where output matching is designed with lumped element π-type network. However, this low Q factor OMN suits broadband. It degrades the output power. The design of GaN-based fully integrated Class-J PA for 5 GHz WLAN 802.11ax systems is presented in Ref.^17^. The performance of this PA can be enhanced further by employing DPD. A Class-J PA designed for X-Band is presented in Ref.^18^, uses the active load modulation technique, facilitates the PA's integrated implementation by eliminating the doubler and filter networks of conventional class-J2 Pas, and achieves high drain-efficiency. But the broad BW is not achieved due to harmonic tuners. The work can be extended further by increasing the BW of the phase shifter, and elements in OMN can be replaced using an active inductor.

### Theory of Class-J operation mode

Class-J is one of the PA modes in which the drain voltage (V_DS_) and drain current (I_D_) can be obtained as half rectified sinusoidal waveforms with a slight overlap between them. The drain current obtained by selecting the Class B biasing point and by considering only fundamental, second harmonics can be expressed as shown in Eq. (1)

1 $${I}_{D}|j\left(\theta \right)=\frac{{I}_{max}}{\pi }+\frac{{I}_{max}}{2\mathrm{cos}\left(\theta \right)}+\frac{2{I}_{max}}{3\pi \mathrm{cos}\left(2\theta \right)}$$

where I_max_ is the peak value of drain current through the transistor. The half-wave rectified sinusoidal drain voltage V_DS_ can be expressed as shown in Eq. (2)

2 $${V}_{DS}| j={V}_{th}+\left({V}_{DD}-{V}_{th}\right)\left(1-\mathrm{cos}\left(\theta \right)\right)\left(1+\alpha \mathrm{sin}\left(\theta \right)\right)$$

where V_DC_ and V_K_ are the biasing voltage and knee voltages of the transistor, respectively,

This drain voltage V_DS_ can be obtained by giving complex inductive and pure capacitive load impedances to Class-J mode PA's transistor at the fundamental and second harmonic frequencies. These optimum load impedances are extracted from Eqs. (1) and (2) are shown in Eqs. (3) and (4)

3 $$Zf0=\frac{\left({V}_{DD}-{V}_{th}\right)\left(1+j\alpha \right)}{Imax/2}={R}_{opt}+j\alpha {R}_{opt}$$

4 $${Z}_{2}f0=-\frac{\left({V}_{DD}-{V}_{th}\right)j\alpha }{\begin{array}{c}2\left(\frac{{2I}_{max}}{3\pi }\right)\end{array}} =-\frac{j3\pi }{8}\alpha {R}_{opt}$$

where R_opt_ is the optimum resistance which can be expressed as shown in Eq. (5)

5 $$R_{{{\text{opt}}}} = {2}\left( {{\text{V}}_{{{\text{DD}}}} - {\text{V}}_{{{\text{th}}}} } \right)/{\text{I}}_{{{\text{max}}}}$$

**Figure 1:**
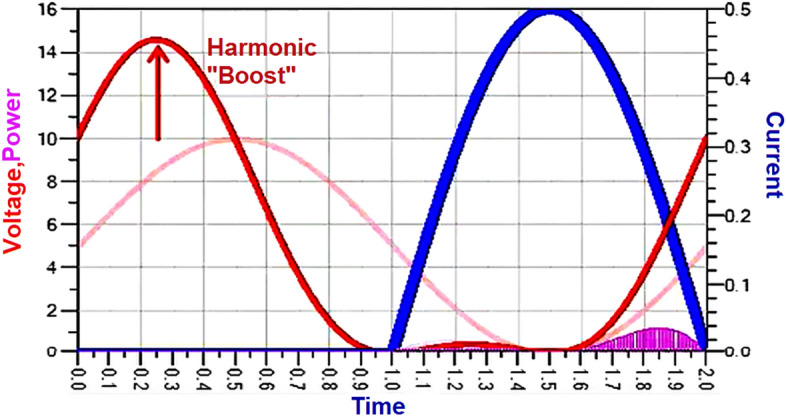
Class-J voltage and current waveforms at the drain of the transistor (V_DS_ and I_D_).

With these optimum load impedances presented, we can observe that the drain voltage (V_DS_) is boosted with a phase shift, as shown in Fig. 1.

Thus, the phase shift and boost in drain voltage (V_DS_) causes a slight overlap with drain current (I_D_), making the Class-J PA highly efficient. Although this waveform shows the feature of switching mode PA, the Class-J mode PA can provide linearity similar to the class-B or AB modes due to its non-switching mode of operation. Unlike class B, there is no need for harmonic traps, making it more appealing for broadband applications.

## Design methodology of Class-J PA using silterra 0.13 µm standard CMOS process technology

**Figure 2:**
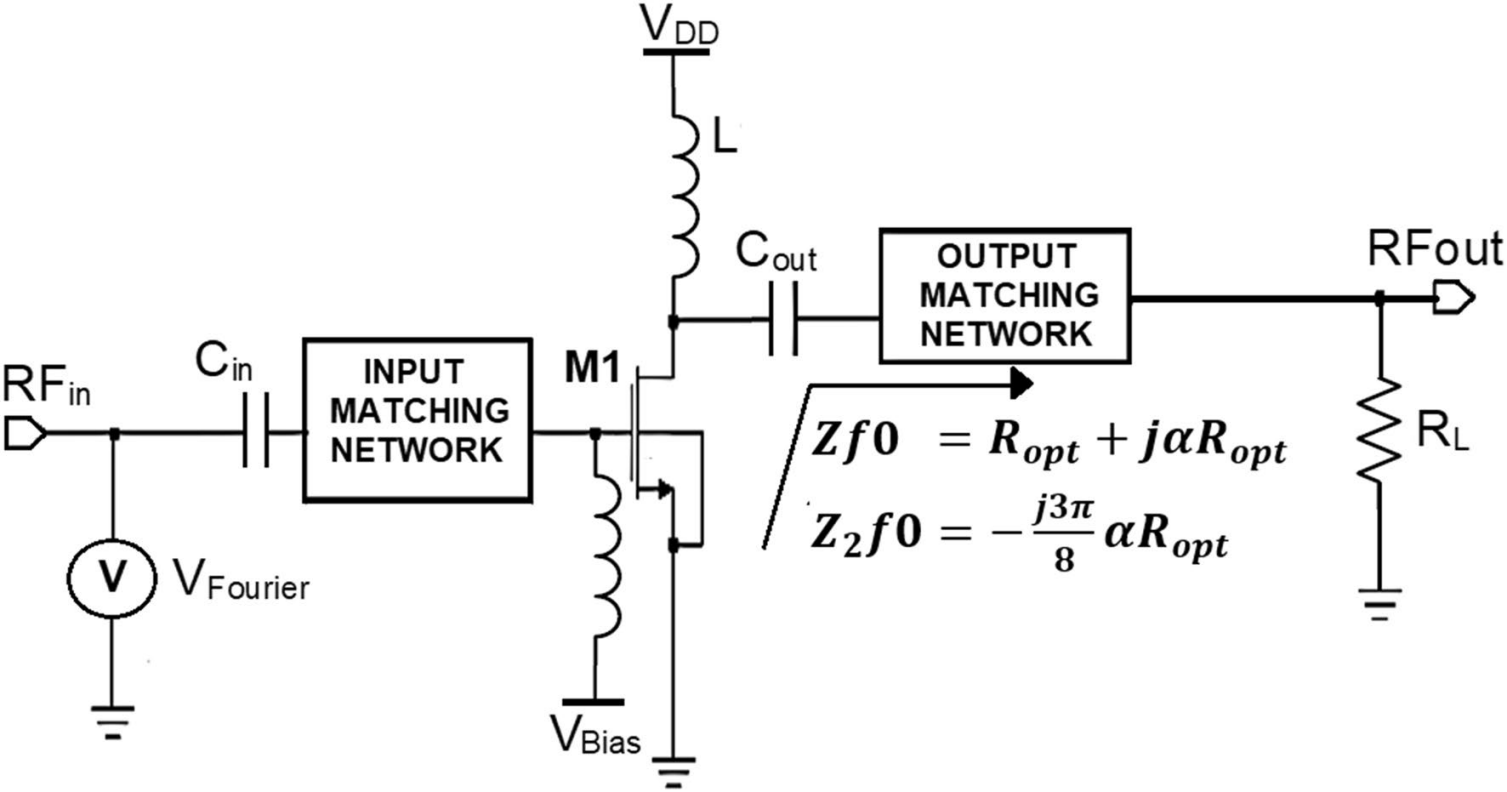
Sample Class-J mode PA topology.

As discussed in “Brief review of switching mode PAs and theory of Class-J PA” among various modes of PA topologies, the Class- J mode PA that was introduced and developed by Cripps in Ref.^12^ is chosen for this research work because of its potential to provide significant enhancement in BW required for the near future 5G wireless communication applications. From the theory of Class-J operation mode explained in “Brief review of switching mode PAs and theory of Class-J PA”. A high-efficiency amplification in broadband can be obtained by presenting fundamental (*Z*_*f0*_) and second harmonic (2*Z*_*f0*_) harmonic optimum load impedances to the NMOS transistor M1 over the desired BW shown in Fig. 2.

**Figure 3:**
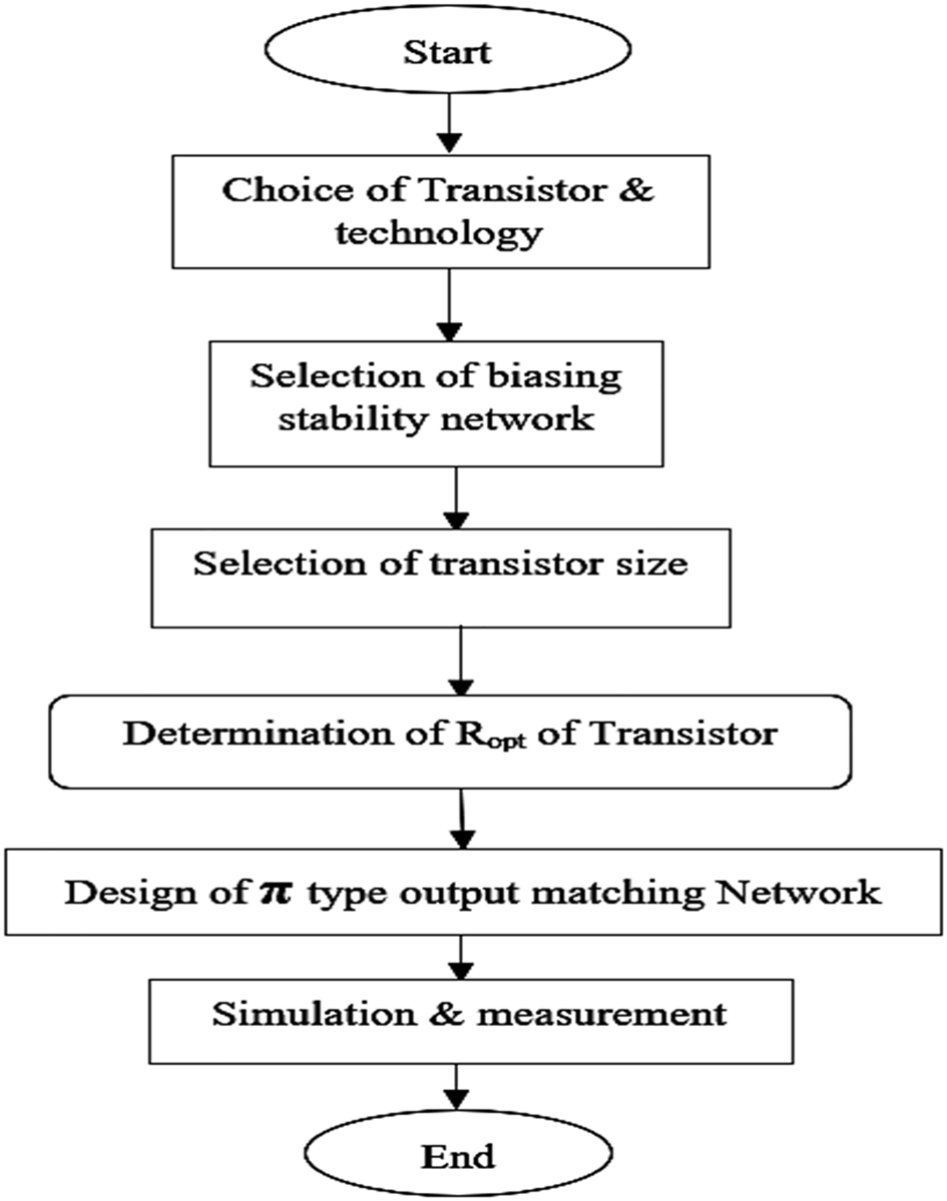
Flow chart of PA’s design methodology.

To design the class J mode PA, a proper OMN must be designed to provide high-efficiency amplification over enhanced BW while maintaining linearity, making the PA suitable for 5G wireless communication applications. The various steps involved in the Class-J mode PA's design methodology is discussed as illustrated in the flowchart shown in Fig. 3.

### Selection of transistor and technology

As per the trend of designing a PA, the mentor graphics design tool is used to simulate the designed PA circuit using siltera-130 nm technology. The nm_hp model NMOS transistor is chosen as it is the available high-power transistor in the silterra 130 nm analog library. The NMOS transistor threshold voltage (V_th_) is determined using its transconductance (gm). To obtain a Class-J PA mode, the load impedances shown in Eqs. (3) and (4) should be presented to this transistor (M1) (i.e., nm_hp) by proper OMN.

### Selection of biasing and stability

The PA design, can choose any kind of supply voltage. As per design idea of the PA, the supply voltage V_DD_ = 5 V and gate voltage V_GS_ $\cong$ V_K_ (threshold voltage) (i.e., with a quiescent bias current of Iq = 2% of Imax) are chosen to bias nm_hp NMOS transistor (M1) used in this work through fixed bias network to operate as a Class-B PA for obtaining a half rectified sinusoidal drain current (I_D_).

One of the most important considerations while designing the PA is that it should be unconditionally stable over the desired frequency range under normal operating conditions. To ensure PA’s stability at low-frequencies, a stabilization circuit consisting of an inductor that also provides the path for DC bias to the gate of transistor M1 is employed. Besides, a parallel RC circuit can stabilize the transistor across the entire range of desired frequencies.

### Selection of transistor size

Generally, the R_opt_ can be calculated theoretically using Eq. (5), which depends on the technology and transistor size (or Pout) chosen, and it can be practically determined by conducting LOADPULL simulations**.** As there is no provision for conducting load-pull simulations in the mentor graphics EDA tool used for this research work, initially, a R_opt_ value = 4.5 Ω which is determined by load-pull simulations, is taken as a reference. This R_opt_ value is obtained by altering transistor size in terms of aspect ratio. Because R_opt_ value can be determined using I_D,_ which depends on the transistor's aspect ratio (W/L). For corresponding V_DD_ and V_GS_ values, the transistor size (i.e., 2 × 5 μm) is selected to realize M_1_to obtain a maximum drain current of about 2.1 A. For V_DD_ = 5 V,V_GS_ = V_K_ = 0.486 V and maximum drain current I_max_ = 2.1A, the optimum resistance obtained using Eq. (5) is R_opt_ = 4.3Ω. Using this R_opt_, the theoretical values of optimal load impedances at fundamental and second harmonic frequencies are determined using Eqs. (3) and (4) as Z_*f0*_ = 4.3 + jα 4.3Ω and Z_*2f0*_ = *− *j 5.2 Ω respectively. To verify these theoretically calculated optimum load impedances and to match the load resistance (RL = 50 Ω) with at least the real part (i.e., R_opt_) of obtained optimum load impedances that have to be presented to the transistor M1 over a wide BW proper OMN has to be designed.

## Output (π)-matching network design

Designing a proper OMN for obtaining Class-J operation mode is challenging as it has to match the output load of RL = 50 Ω with the optimum load impedances **Z**_***f0***_ and **Z**_***2f0***_ of transistor M1. The desired matching in wideband can be achieved by realizing a high-order OMN, which may need a minimum of two series inductors when lumped elements are used, but due to the losses of integrated inductors used in OMN, the power output and the efficiency of PA will be reduced. This reduction is higher than the reduction caused by the load impedance mismatch. Out of different methods for realizing OMN such as lumped element matching (i.e., L, T,π topologies) and distributed matching (i.e., using transmission line networks), initially a simplified lumped element π-type OMN with one inductor and two capacitors is chosen for this Class-J PA design because the distributed matching using transmission lines occupies a larger area which in turn increases the cost and there is an idea to replace the lumped passive elements of the π- matching network with active elements as further progress of this PA design.

**Figure 4:**
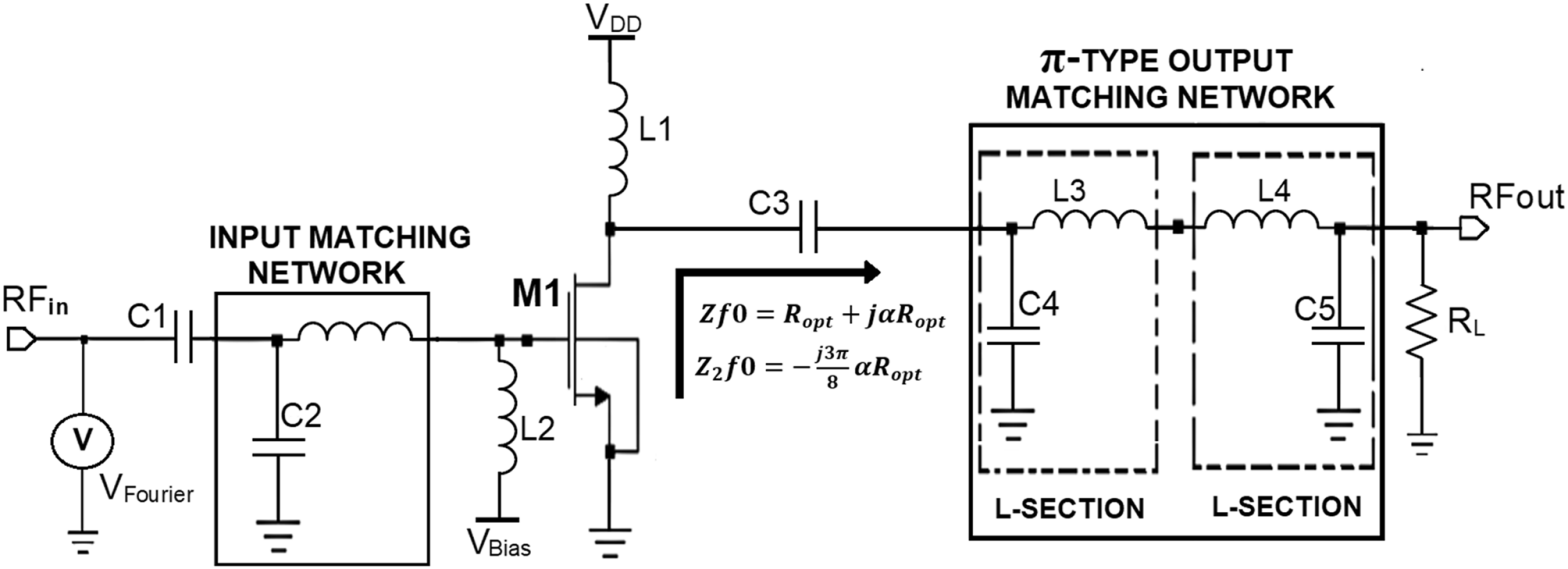
Sample Class-J mode PA topology with π**-**matching network.

The π-type OMN chosen in this work is designed to operate over a BW of 500 MHz with the center frequency of 5 GHz (i.e., sub 6 GHz) as the Class-J PA has to be operated at 5G frequency. The quality factor can be calculated with the desired BW and center frequency as Q = f/BW. This work uses the general design procedure of the π- matching network in which the π section will be split into two back-to-back connected L -sections, as shown in Fig. 4.

So, the elements of π-type OMN use the formulae of the L-type matching network. Initially, as π-section is split into 2 back-to-back connected L-sections. The load resistance seen by the first L -Section is assumed as Rv [i.e., R_V_ = R_L_], which can be obtained in terms of R_opt_ as shown in Eq. (6).

6 $$R_{{\text{v}}} = {\varvec{R}}_{{{\varvec{opt}}}} / (Q^{2} + 1)$$

The Q is the quality factor expressed as f /BW, where f and BW are the desired input frequency and bandwidth.

Now with values of Q and R_V_ the inductor L_1_ value can be calculated as shown in Eqs. (7) and (8).

7 $$XL_{1} = QR_{L} = QR_{{\text{V}}} \Omega$$

8 $$L_{1} = X_{{{\text{L1}}}} /2{\pi f}$$

The capacitor C_1_ value can be calculated as shown in Eqs. (9) and (10).

9 $$XC_{1} = {\text{R}}_{{{\text{OPT}}}} /{\text{Q}}$$

10 $$C_{1} = 1/2\pi {\text{fX}}_{{{\text{C1}}}}$$

In the second L-section, L_2_ and C_2_ can be calculated by assuming the value of [R_V_ = R_opt_] with the load resistance R_L_ of 50Ω.

The L-network relationship can define the quality factor Q of this second L section, as shown in Eq. (11).

11 $$Q_{{{\text{new}}}} = \sqrt {\left( {R{\text{L }}/ R{\text{V}}} \right) {-}1]}$$

The inductance L2 value can be calculated as shown in Eqs. (12) and (13).

12 $$XL_{2} = Q_{{{\text{new}}}} R_{{{\text{OPT}}}}$$

13 $$L_{2} = ({\text{X}}_{{{\text{L}}2}} )/2\pi {\text{f}}$$

The capacitor C_2_ can be calculated as shown in Eqs. (14) and (15).

14 $$XC_{2} = {\text{R}}_{{\text{L}}} /{\text{Q}}_{{{\text{new}}}}$$

15 $$C_{2} = 1/2\pi {\text{fX}}_{{{\text{C2}}}}$$

The capacitance C_1_ of the π- matching network should be chosen carefully so that it has to take output parasitic capacitance C_DS_ of the transistor into account because this capacitance at the higher-order harmonic frequencies is considered as a short circuit. Once the OMN is designed, the capacitive reactance to the load line resistance ratio [X_CDS_/R_L_] must be calculated. Suppose this ratio is (< =) 1, then the matching network design is considered ideal. But, depending on the device technology used and the frequency, this ratio can also be above the unity.

## Simulation results

**Figure 5:**
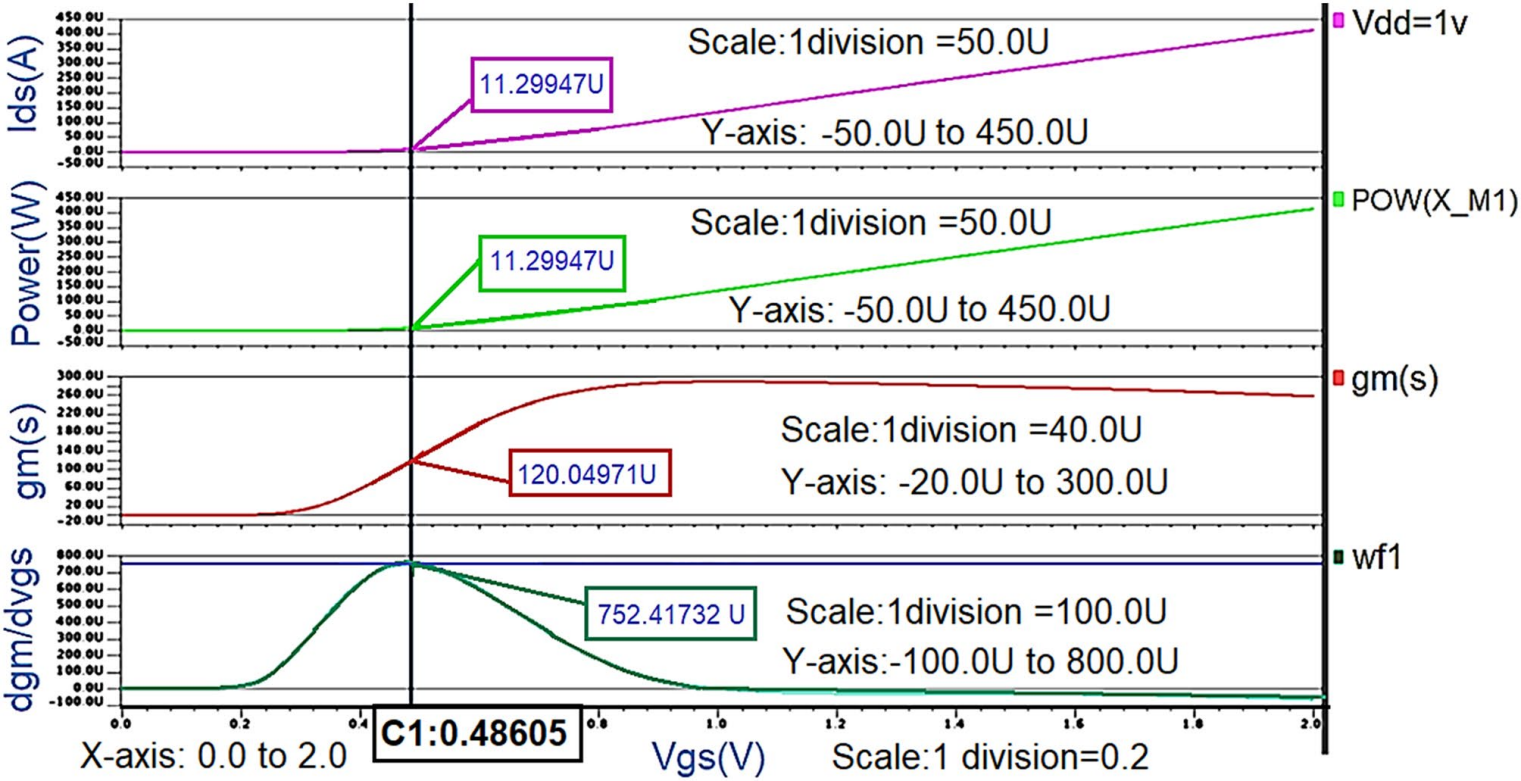
V_th_ of nm_hp model NMOS transistor.

As explained in “Design methodology of Class-J PA using silterra 0.13 µm standard CMOS process technology” and “Output (π)-matching network design”, the Class-J PA is simulated using nm_hp model NMOS transistor from 130 nm Silterra analog library in the MENTOR GRAPHICS EDA tool as the main transistor M1. “Design methodology of Class-J PA using silterra 0.13 µm standard CMOS process technology” discusses the theoretically calculated reference R_opt_ value using Eq. (5) is 4.3 Ω. To verify this theoretically calculated reference R_opt_, the values of threshold voltage (V_th_) and the peak drain current (I_Dmax_ ) of the chosen NMOS transistor (i.e., nm_hp model NMOS) need to be estimated. The transistor's threshold voltage (Vth) can be extracted from the value of gm, which is a figure of merit to indicate how well the device converts the voltage to current. It can be represented as dI_D_ /dV_GS_.Using the waveform calculator utility of the EZ wave plot in the MENTOR GRAPHICS EDA tool, the drain current (I_DS_) is differentiated concerning V_GS_ to get gm. After obtaining the gm value, it is differentiated with respect to V_GS_ to extract threshold voltage (Vth). The gate voltage V_GS_ at which the maximum dgm /dVGS value is considered the threshold voltage (Vth) is shown in Fig. 5.

**Figure 6:**
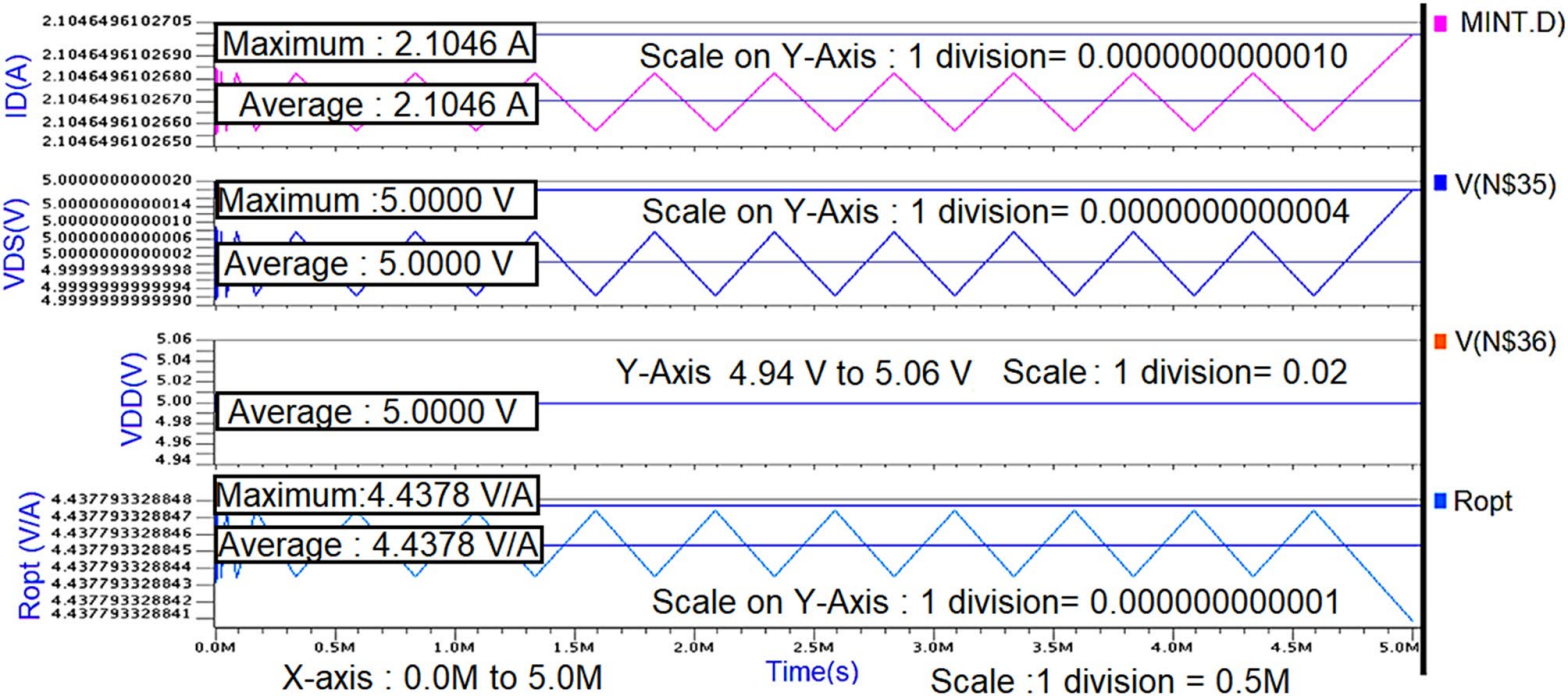
R_opt_ obtained from the waveform calculator.

After extracting the threshold voltage(V_th_), the Class-B biasing point is chosen to obtain the maximum drain current I_Dmax_ corresponding to the reference Ropt. The transistor's sizing is selected as explained in “Selection of transistor size”. The I_Dmax_ obtained for the transistor's biasing and sizing and the corresponding R_opt_ value calculated using the waveform calculator are almost the same as the theoretically calculated values, as shown in Fig. 6.

**Figure 7:**
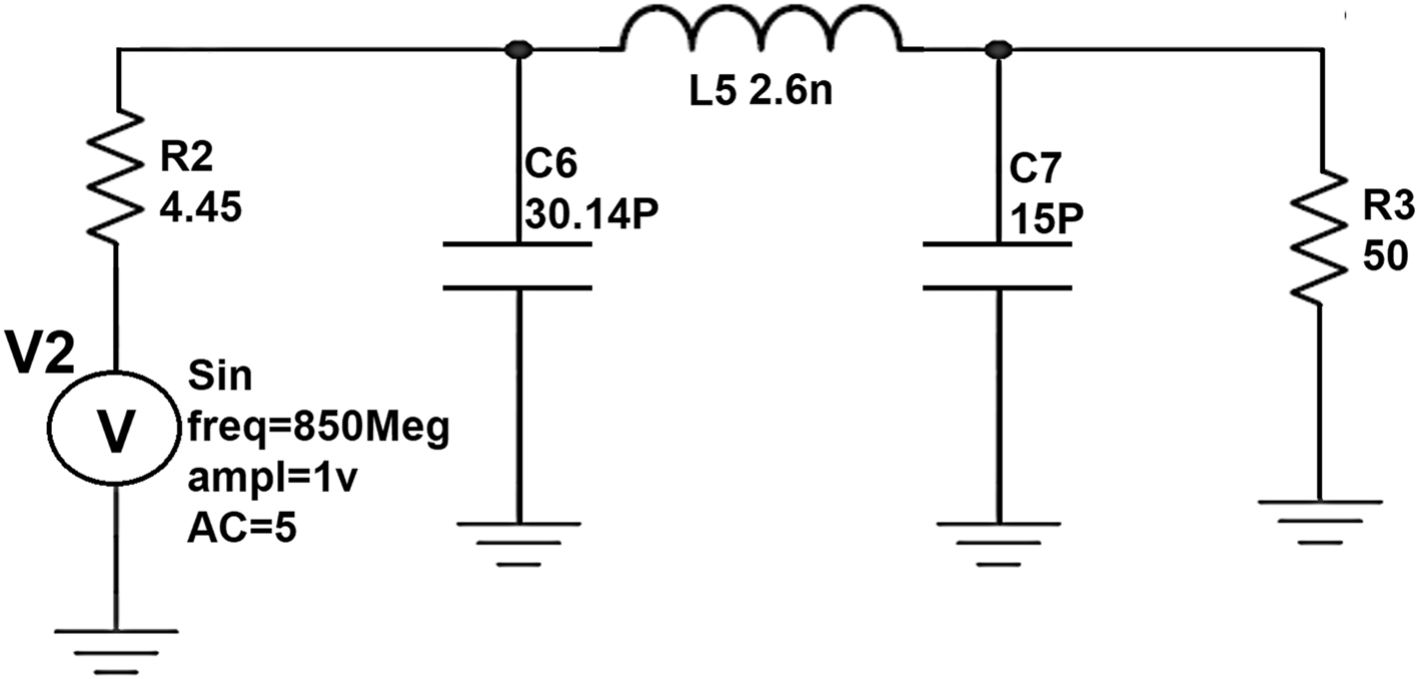
Schematic circuit of the π-type matching network.

**Figure 8:**
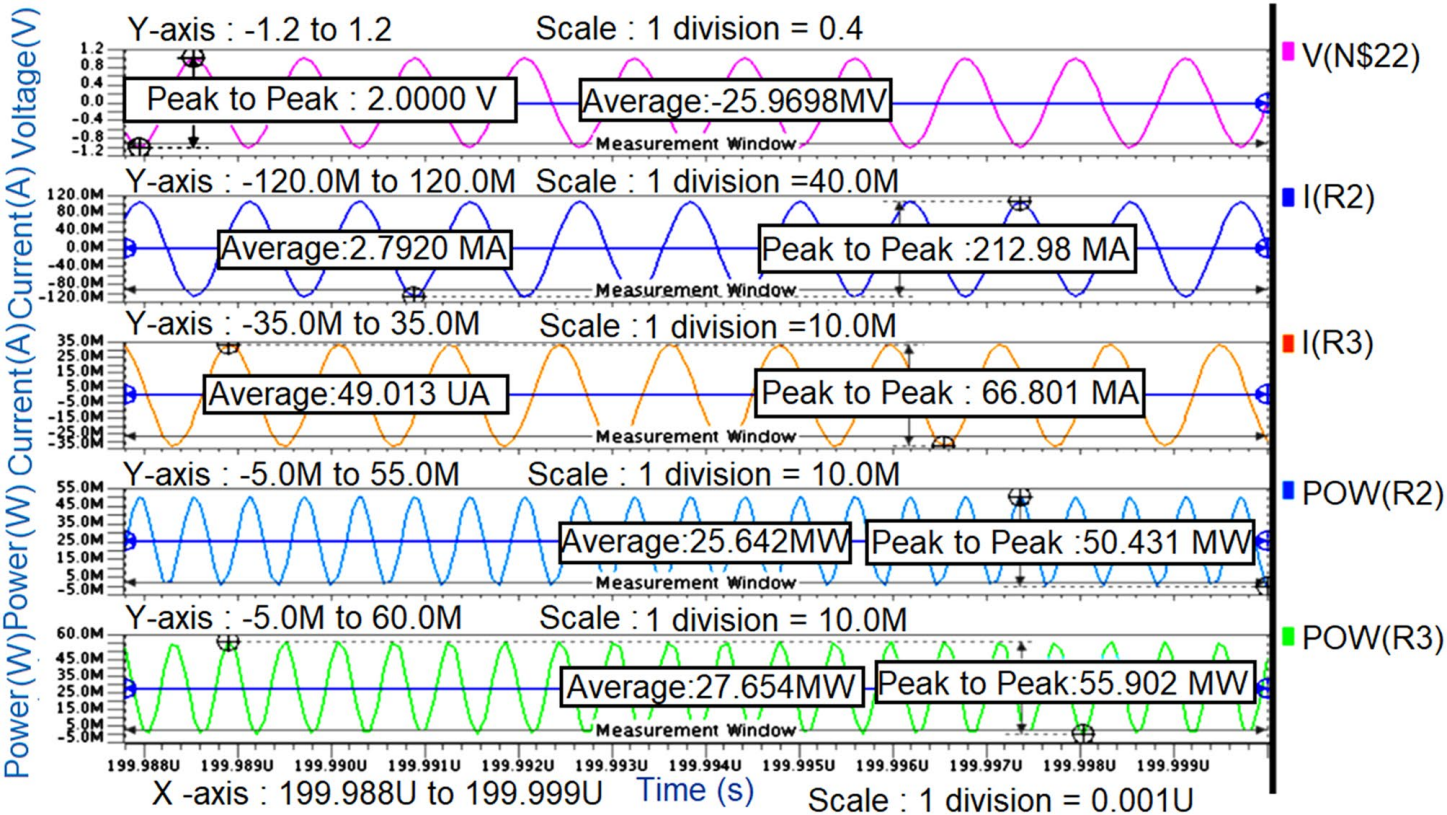
Transient response of the π-type matching network.

Initially by taking the Ropt obtained from the waveform calculator as reference (i.e., R2), a π-matching network with a sinusoidal input source at 850 MHz frequency is designed using the design equations discussed in “Output (π)-matching network design” to verify whether the maximum power is getting a transfer from the source to load. The schematic circuit of the π-matching network to match R_L_ (i.e., R_3_) with R_opt_ (i.e., R_2_) and its transient response are shown in Figs. 7 and 8.

From the graph shown in Fig. 8, it can be observed that for a sinusoidal input source with a peak to peak of 2 V (i.e., the amplitude of 1 V) at 850 MHz frequency, the power measured across load resistance R_3_(i.e., R_L_) is 27mW which is nearly the same as the power measured across the source resistance R_2_ (i.eR_opt_) is 26mW even though the average current across load resistance is less(i.e., 49µA) compare to the average current across source resistance (i.e., 2.8 mA) which means that the maximum power is transferred to load resistance from source resistance by means of a π-type matching network as shown in Fig. 7.

**Figure 9:**
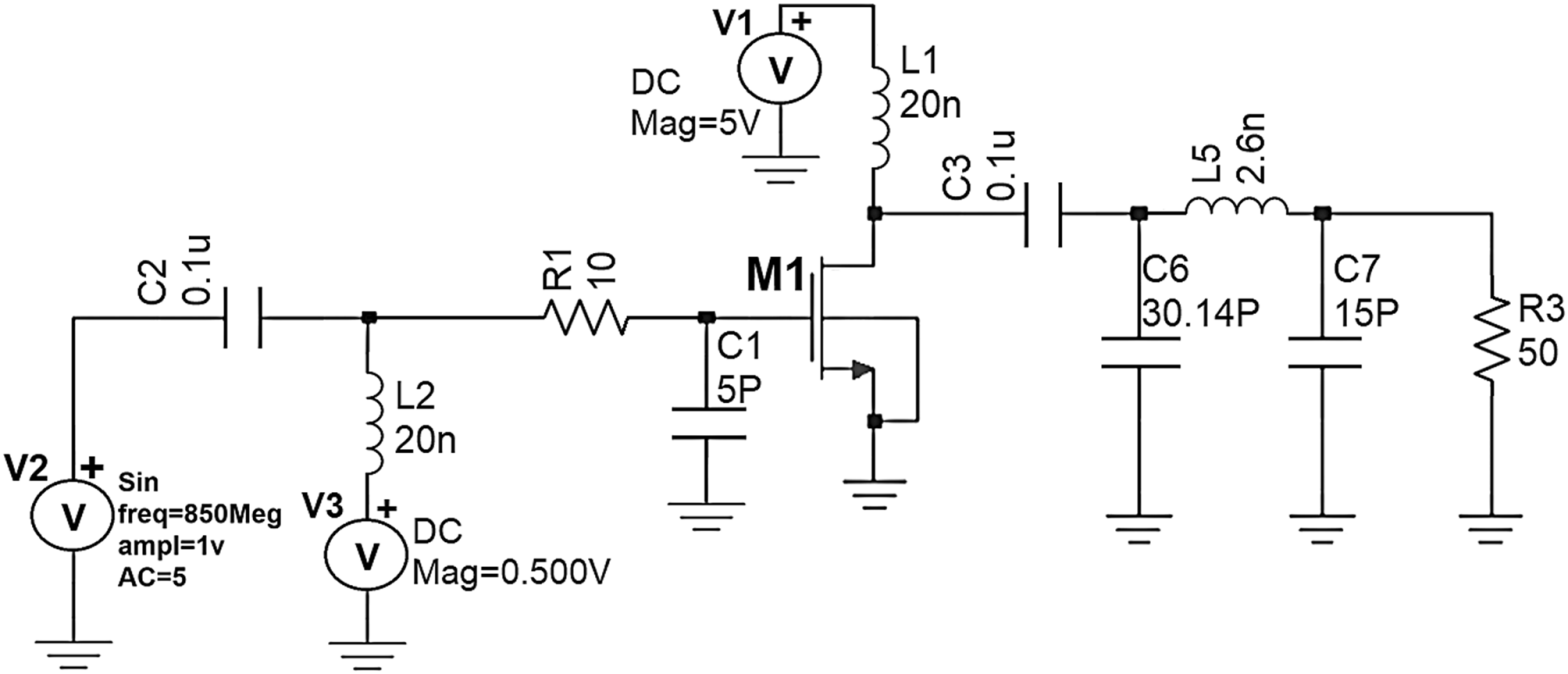
The schematic circuit of Class-J PA with a sinusoidal input source at 850 MHz.

**Figure 10:**
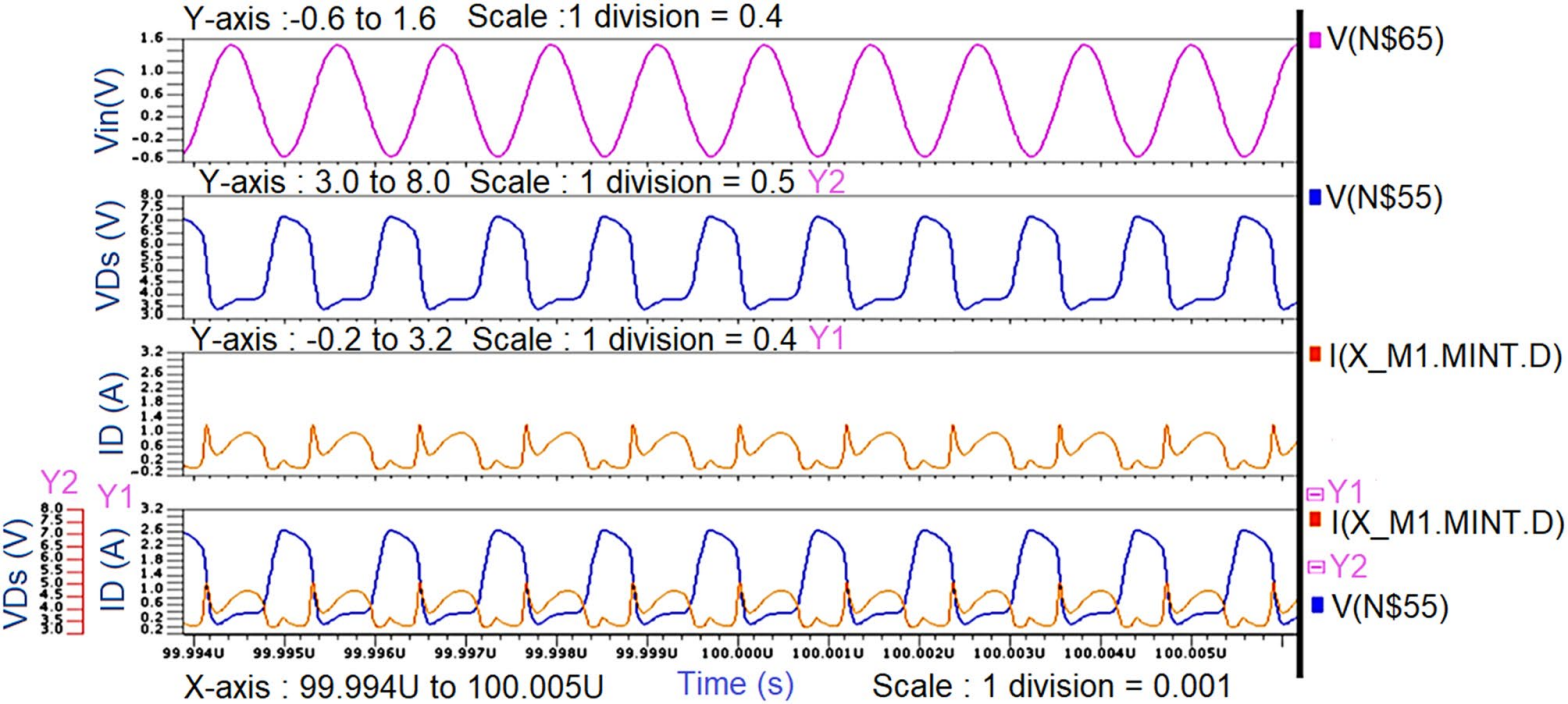
Transient response of Class-J PA with sinusoidal input source at 850 MHz.

The Class-J PA is initially designed by realizing this π-matching network with a sinusoidal input source at 850 MHz. The schematic Class-J PA circuit and its transient response are shown in Figs. 9 and 10.

The simulated transient response waveforms shown in Fig. 10 are obtained by presenting the required optimum load impedances in terms of R_opt_ at fundamental and second harmonic frequencies to the transistor through the π-type matching network.

**Figure 11:**
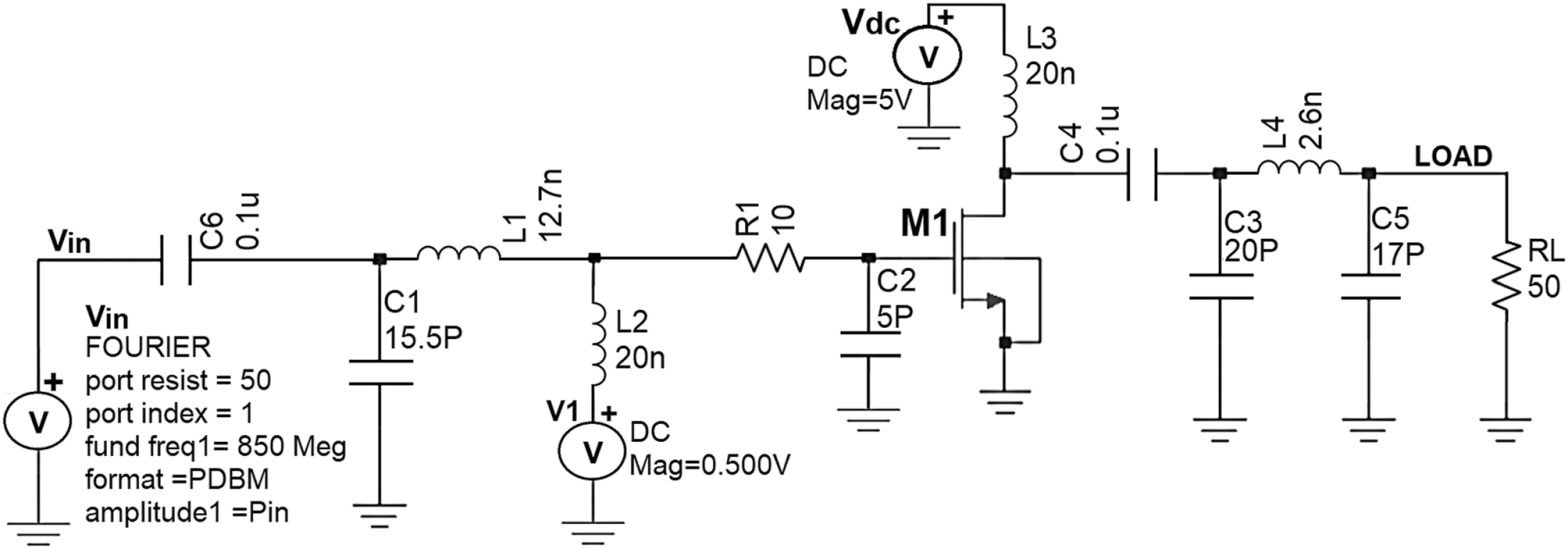
Schematic circuit of the Class J PA with Fourier input source at 850 MHz.

**Figure 12:**
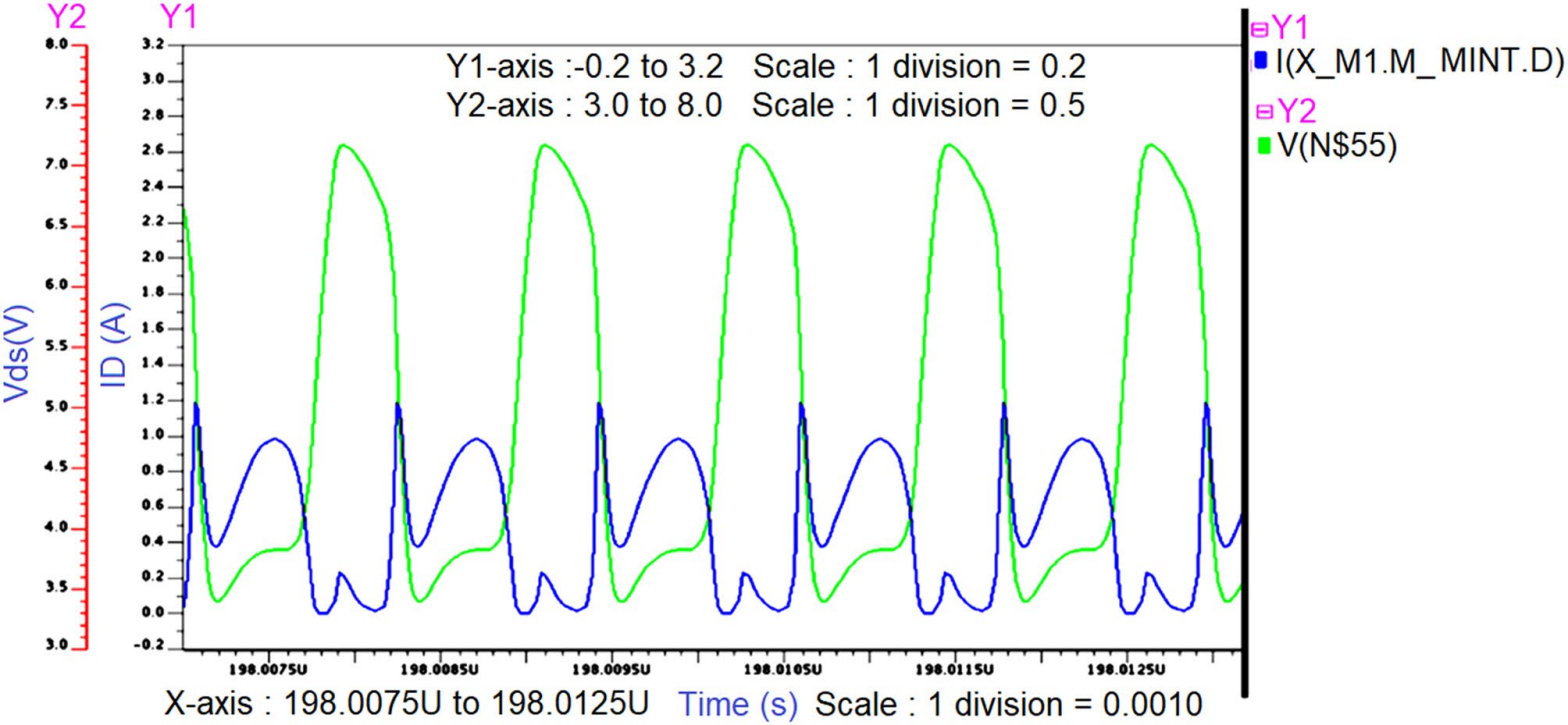
Intrinsic voltage and current waveforms at the drain of Class-J PA (V_DS_ and I_D_) with Fourier input source at 850 MHz.

The Class -J PA is designed by realizing this π-matching network with a Fourier input source at 850 MHz as RF input. The schematic Class-J PA circuit and its transient response are shown in Figs. 11 and 12.

The simulated voltage and current waveforms at the drain of the transistor (V_DS_ and I_D_) are shown in Fig. 12. We can observe that nearly half rectified drain current (I_D_) is obtained due to Class B biasing. The drain voltage (V_DS_) is obtained as a half-rectified sine wave with the harmonic boost and phase shift by presenting optimum load impedances to transistor M1 at fundamental and second harmonic frequencies, which exhibits the Class-J PA mode as explained in “Brief review of switching mode PAs and theory of Class-J PA”.

**Figure 13:**
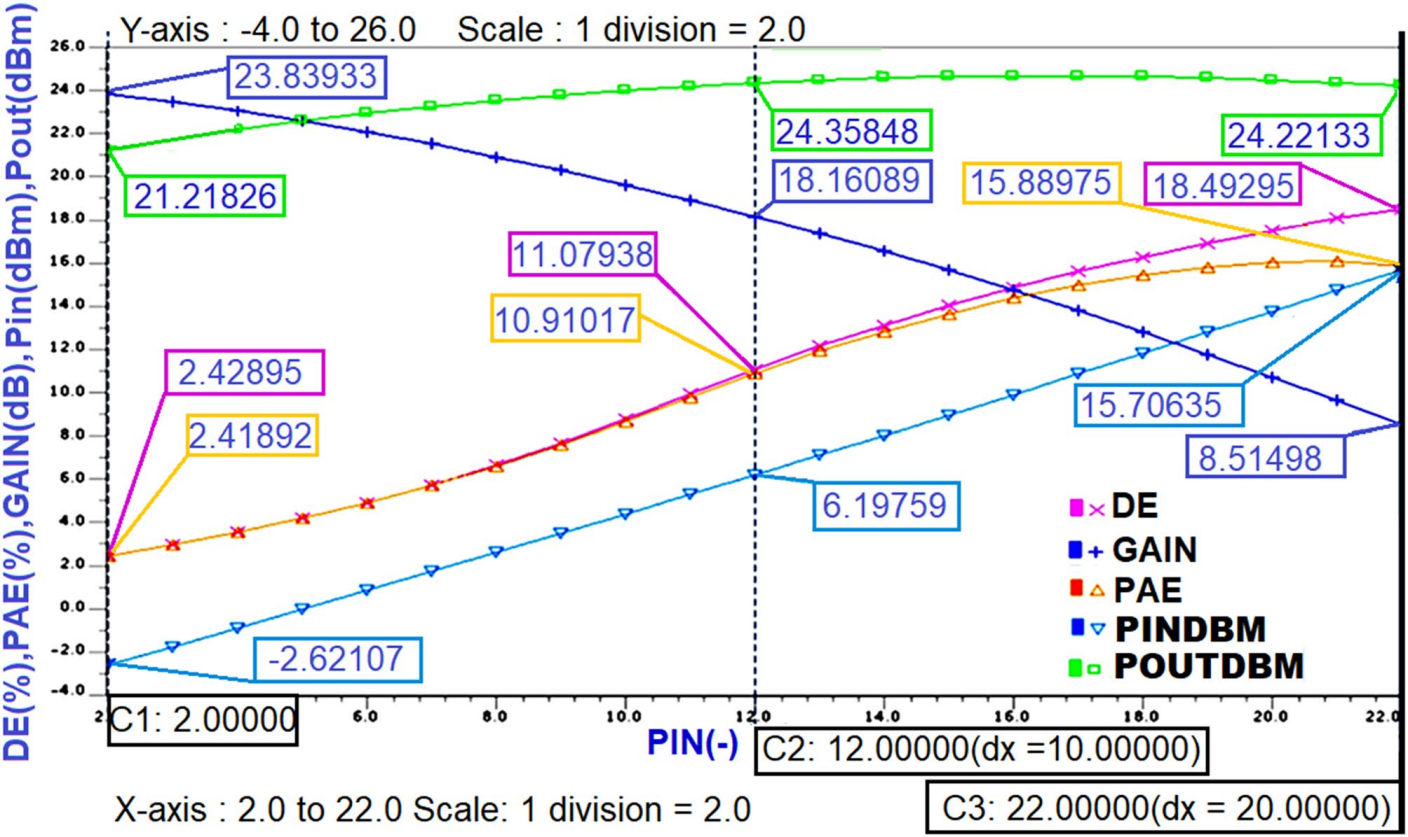
The performance parameters of Class-J PA with RF input at 850 MHz.

After obtaining the expected Class-J mode voltage and current waveforms, the important performance parameters such as power Gain, Pin dBm, power Output (Pout dBm), (PAE), and Drain Efficiency (DE) of designed PA needs to be obtained. The extraction of these parameters is performed for each Pin value swept during parametric sweeping using Steady-state (SST) analysis. Theoretically, the (DE) can be calculated as output power delivered to load divided by DC power consumed by the PA, (PAE) can be calculated as the difference between output power and input power divided by the total DC power consumption, and the gain can be calculated by subtracting the input power from the output power when measured in dB. These parameters are obtained in graphical representation by typing these theoretical formulae as. EXTRACT commands in the SST analysis command window in the Mentor graphics EDA tool. The steady-state analysis results of performance parameters for the Class-J PA designed at 850 MHz centre frequency is shown in Fig. 13.

**Figure 14:**
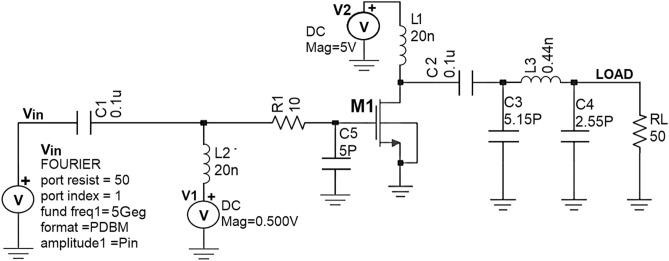
Schematic circuit of the proposed Class-J PA with Fourier input source at 5 GHz.

**Figure 15:**
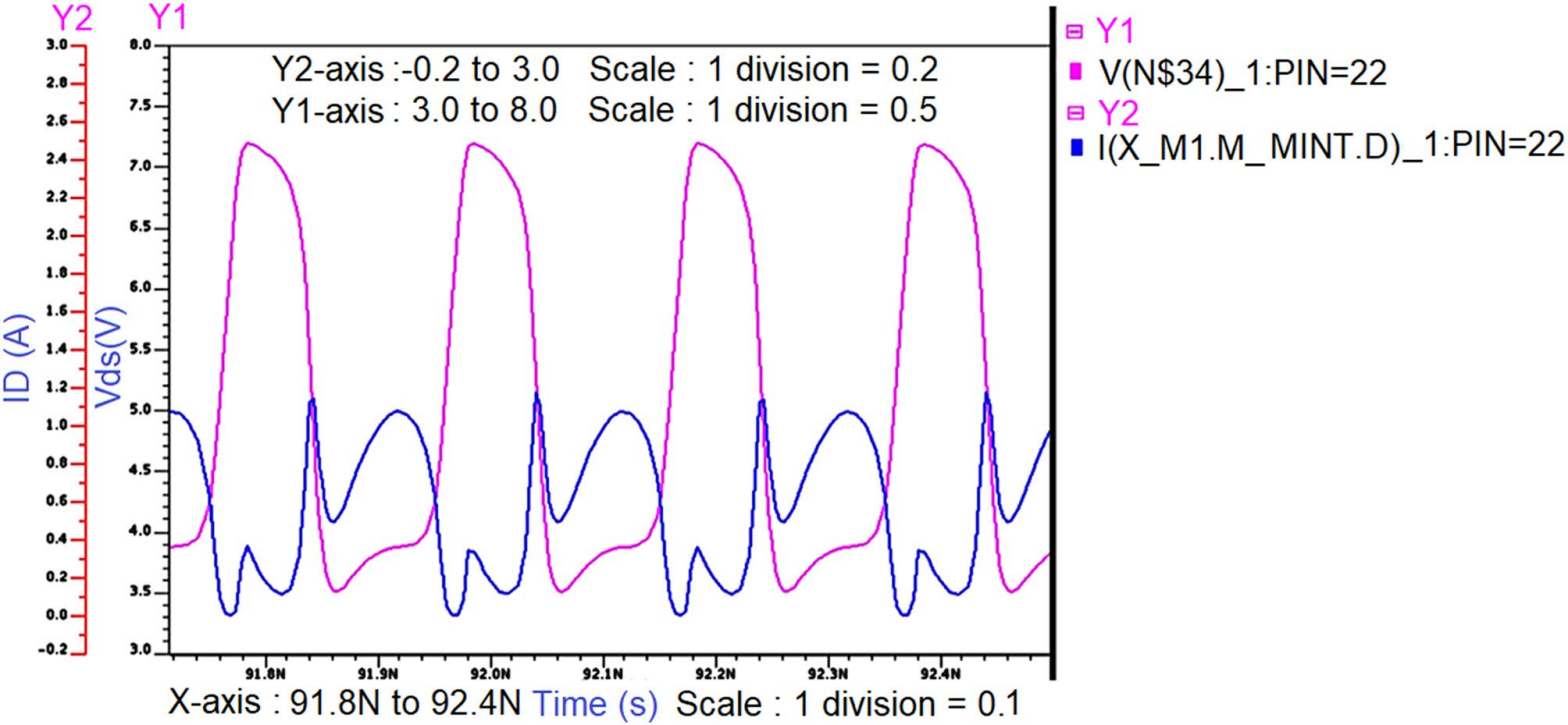
Intrinsic voltage and current waveforms at the drain of proposed Class-J PA (V_DS_ and I_D_) with Fourier input source at 5 GHz.

As this research work mainly focuses on PA design that suits 5G wireless communication applications, the same Class-J PA is designed by realizing this π-matching network with Fourier input source at a 5-GHz frequency (i.e., sub-6-GHz suits for 5G technology) as RF input. The schematic Class-J PA circuit and its transient response are shown in Figs. 14 and 15.

The waveforms of voltage and current at the drain of the transistor (V_DS_ and I_D_) obtained from the simulation of Class-J PA with the proper realization of the π-matching network at 5 GHz frequency will exhibit a phase overlap between them as shown in Fig. 15, and these waveform shapes are similar to the waveforms reported in Ref.^10^, which is the key feature of the Class-J mode PA as explained in “Brief review of switching mode PAs and theory of Class-J PA”.

**Figure 16:**
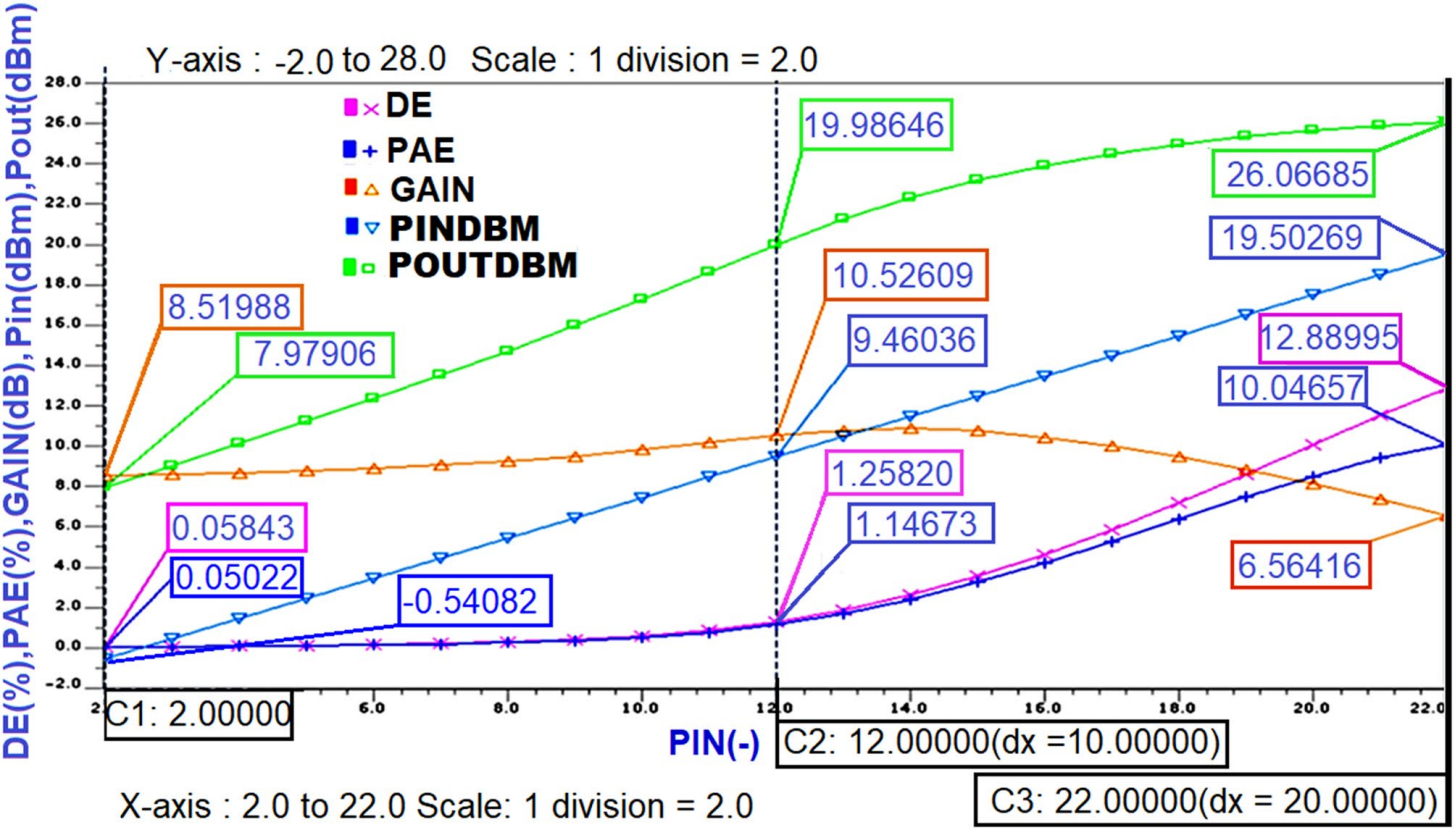
The performance parameters of Class-J PA with RF input at 5 GHz.

The important steady-state analysis results of performance parameters of the Class-J PA designed at 5 GHz centre frequency that was obtained in the same manner as the Class-J PA designed at 850 MHz centre frequency are represented in Fig. 16.

**Figure 17:**
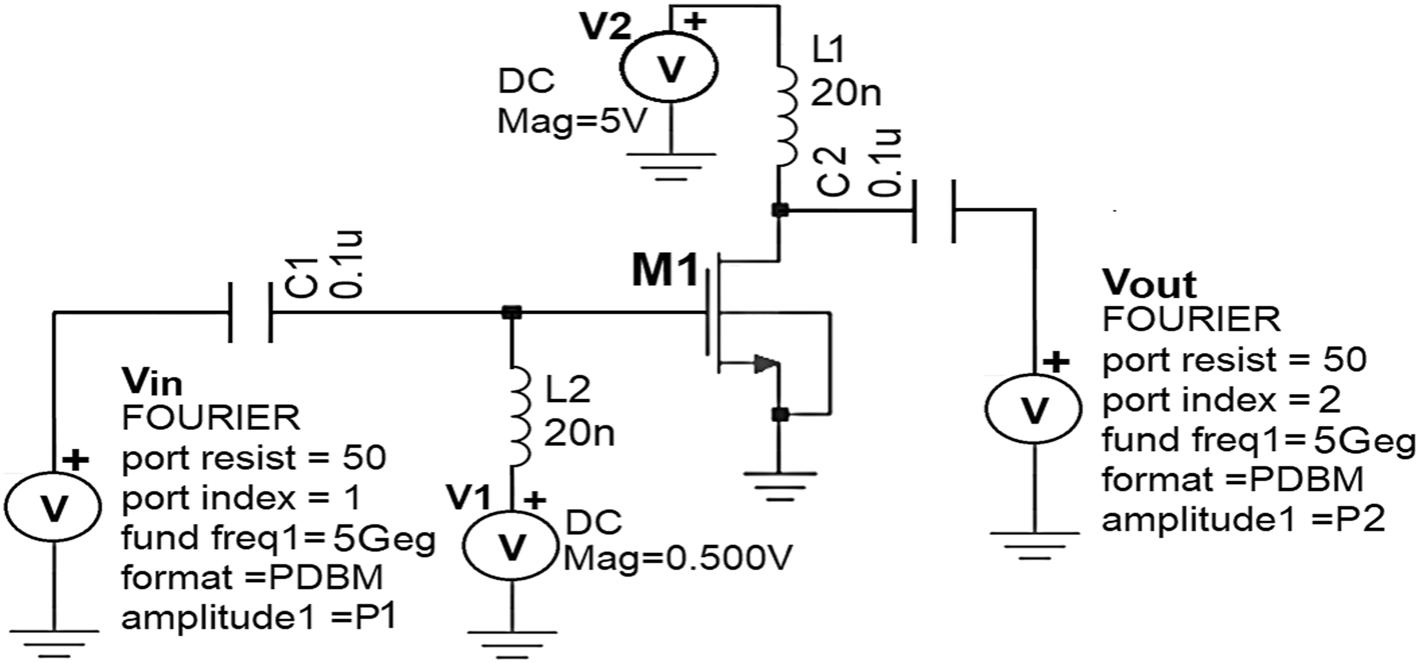
S-Parameter and Z_out_ extraction of nm_hp NMOS transistor at 5 GHz frequency.

As an alternate approach to find the accurate R_opt_ value, the nm_hp NMOS transistor’s output impedance (Z_out_) of the schematic circuit shown in Fig. 17 is calculated in terms of S-parameters using the .def wave commands using SST analysis.

**Figure 18:**
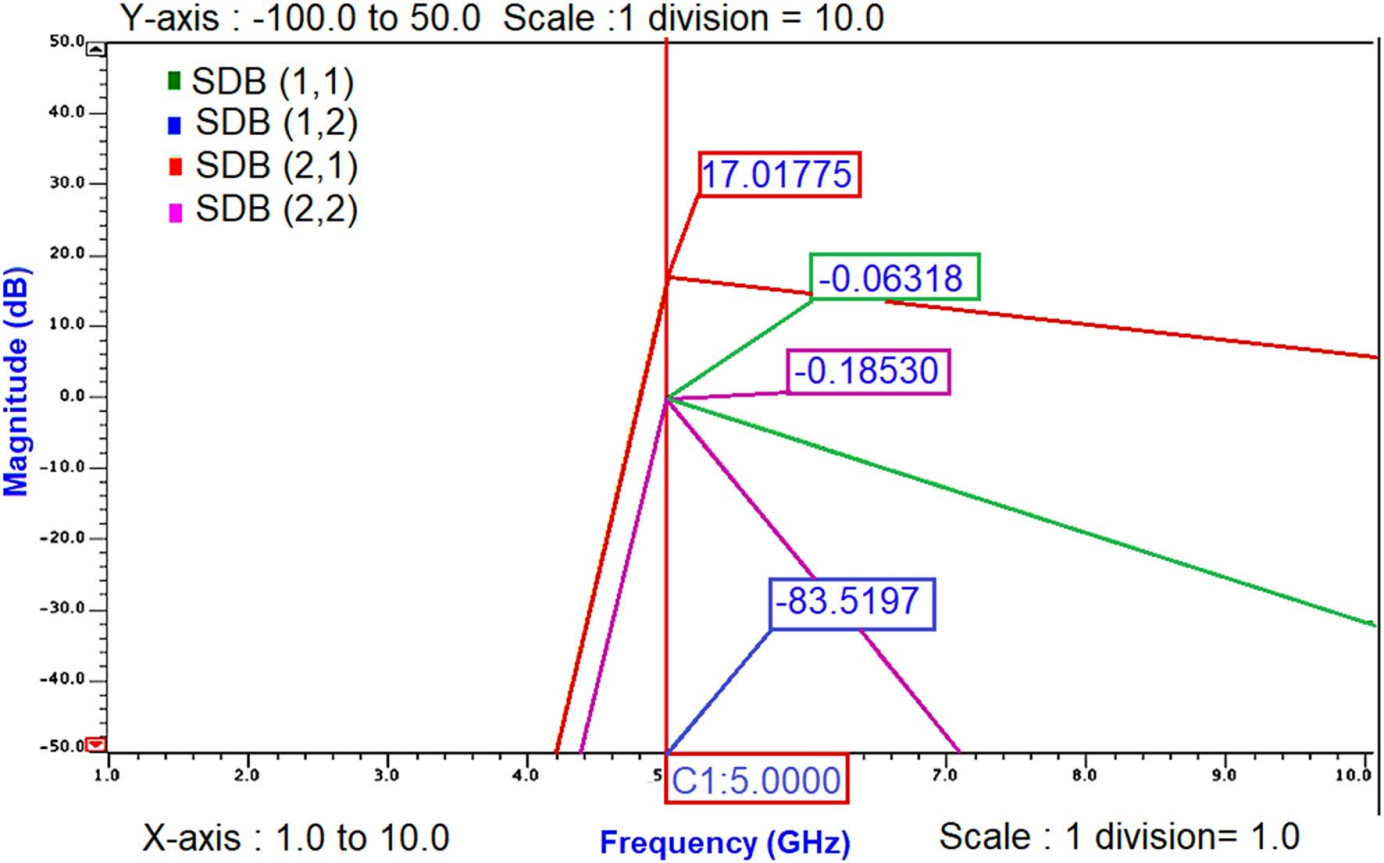
S-parameters of nm_hp NMOS transistor at 5 GHz frequency.

The maximum small signal gain S_21_ of 17 dB at 5 GHz is obtained from the S-parameter simulations as shown in Fig. 18.

**Figure 19:**
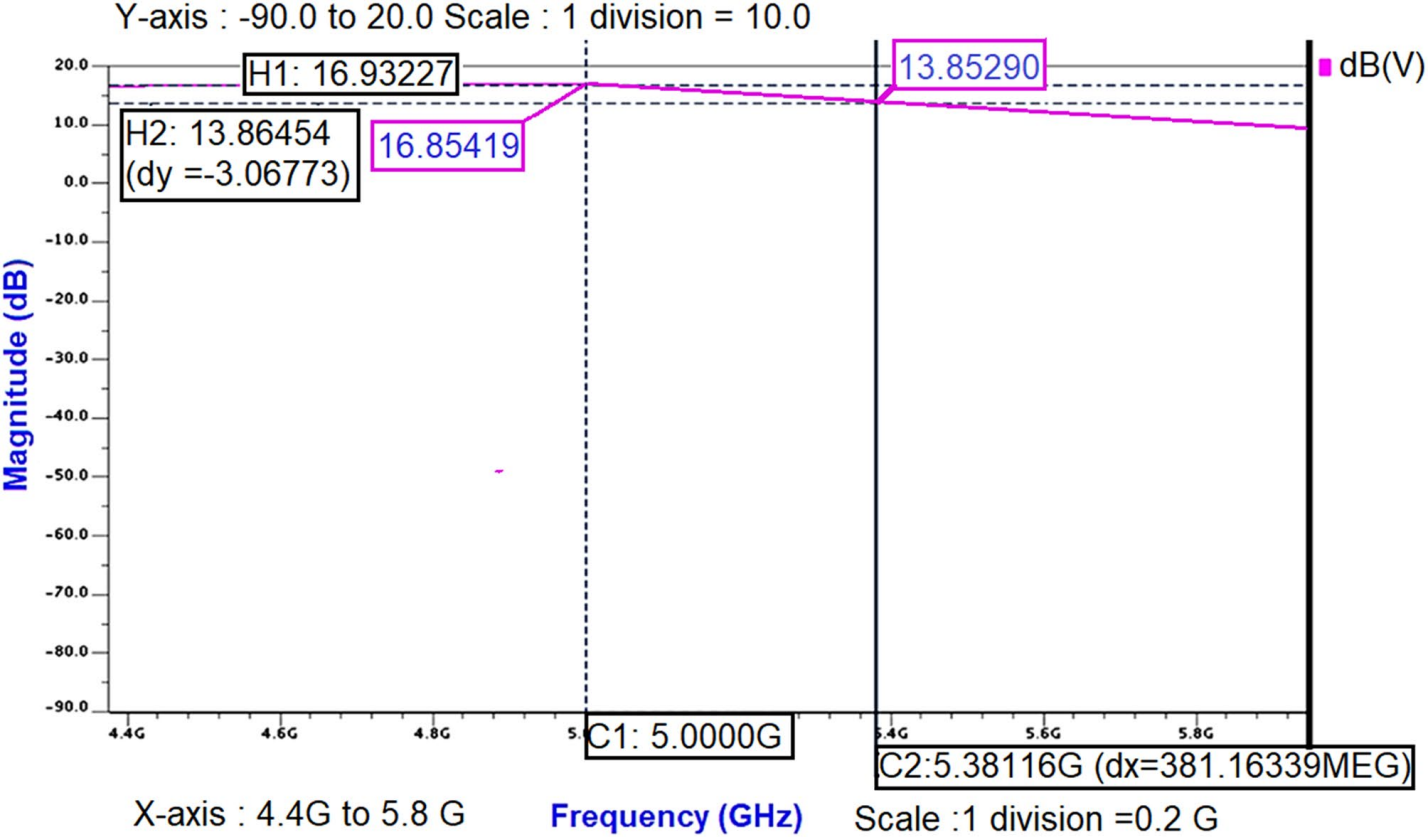
The small-signal gain obtained from AC analysis of Class-J PA.

To validate the small-signal gain S_21_ obtained from .def wave commands, AC analysis of Class-J PA was performed and noticed that the small-signal gain around 16.85 dB is obtained over the frequency 4.3–5.6 GHz, as shown in Fig. 19.

**Figure 20:**
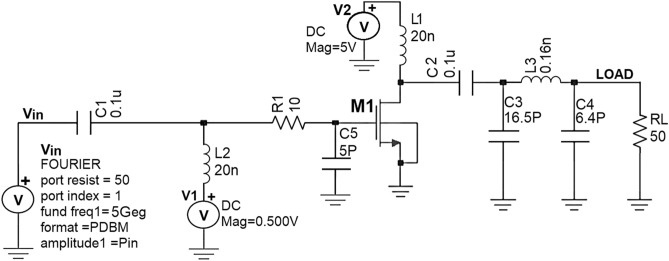
Schematic circuit of the Class-J PA with Fourier input source at 5 GHz.

**Figure 21:**
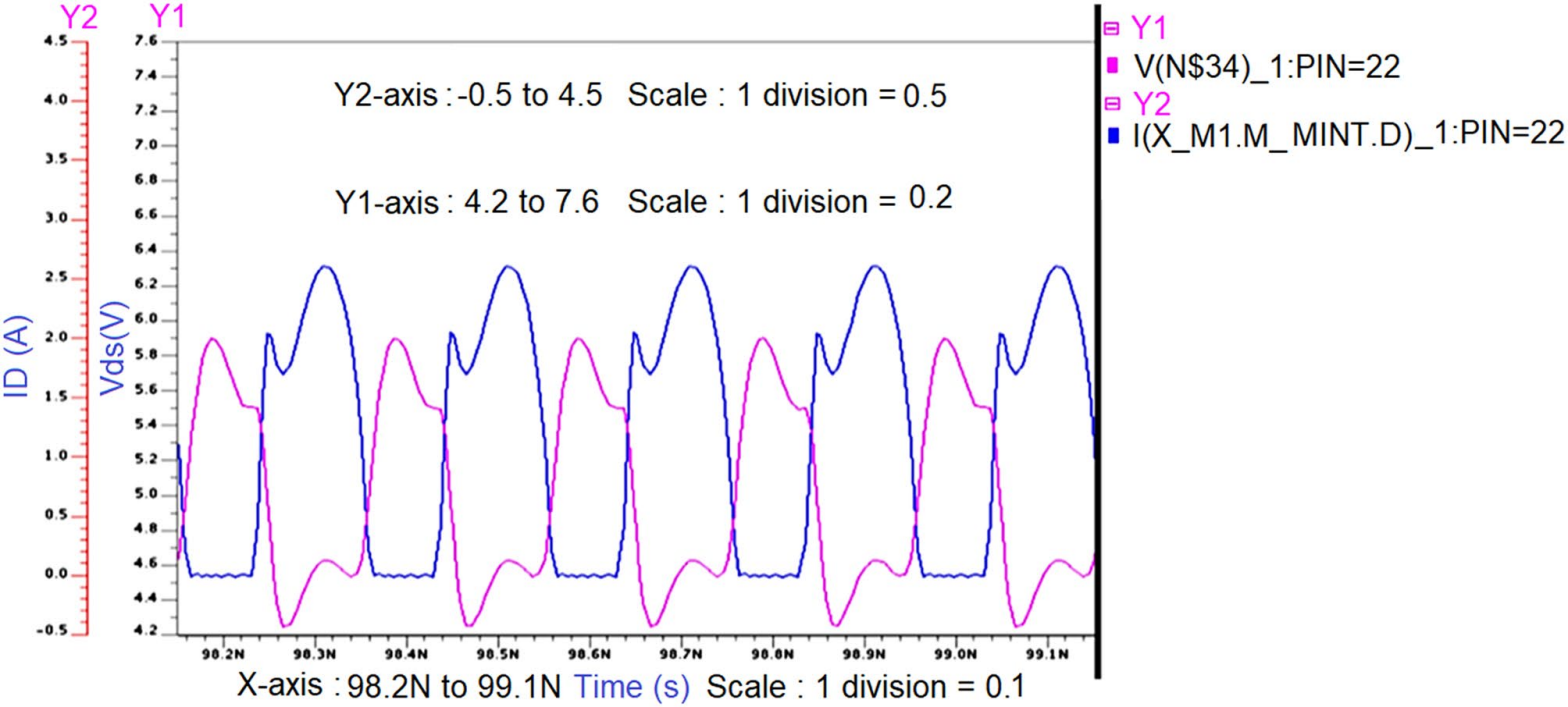
Intrinsic voltage and current waveforms at the drain of Class-J PA (V_DS_ and I_D_) with Fourier input source at 5 GHz.

By considering the output impedance (Z_out_) of the nm_hp NMOS transistor obtained using the .def wave commands from SST analysis as the R_opt_ value, the Class J PA is designed by realizing this π-matching network with Fourier input source at a 5-GHz frequency. Figures 20 and 21 show the schematic circuit and its transient responses.

The simulated voltage and current waveforms at the drain of the transistor (V_DS_ and I_D_) are obtained similarly as explained for Fig. 15 are shown in Fig. 21, which exhibits the key feature of Class-J PA mode as discussed in “Brief review of switching mode PAs and theory of Class-J PA”.

**Figure 22:**
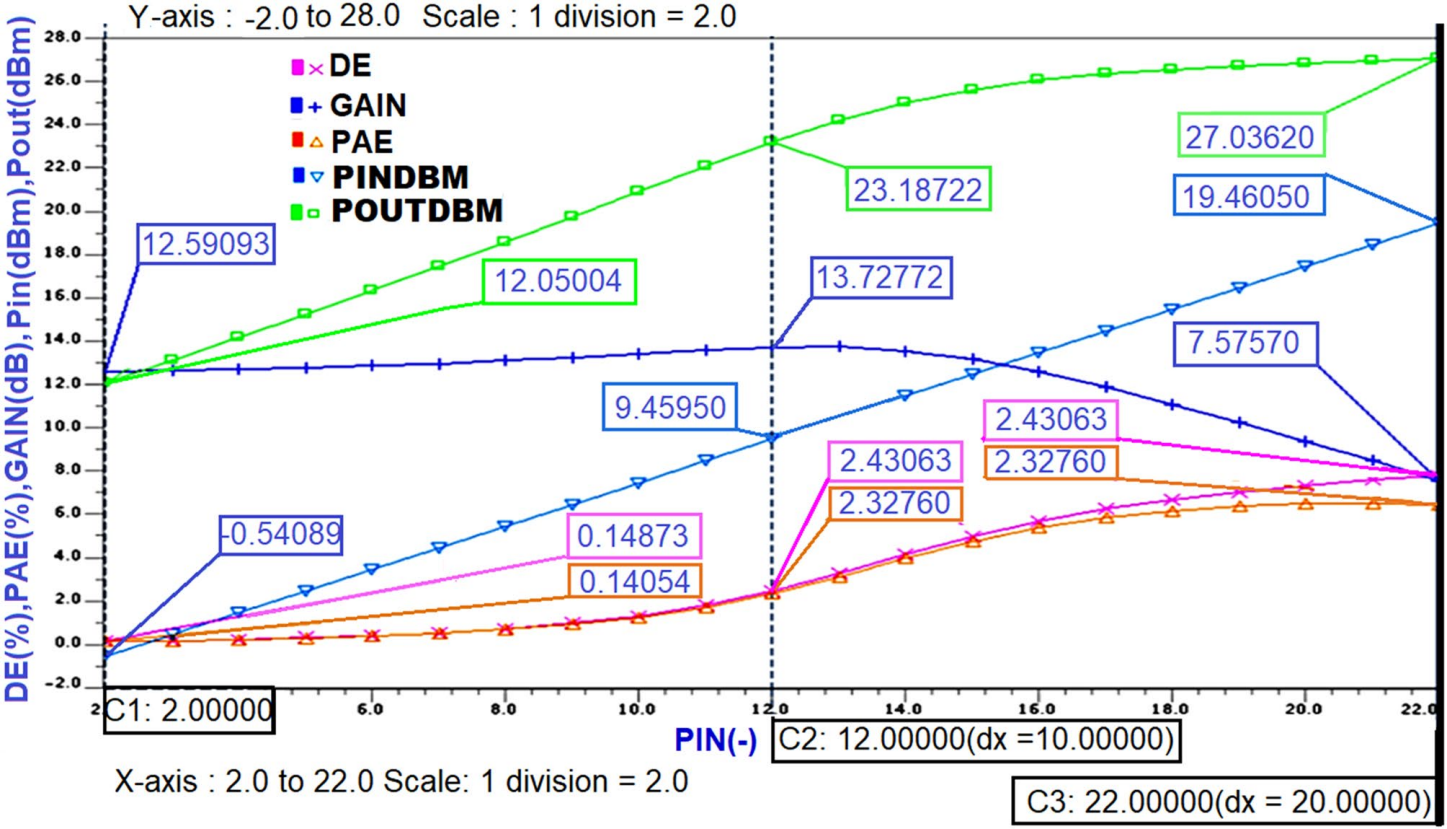
The performance parameters of Class-J PA with RF input at 5 GHz.

The important performance parameters obtained from steady-state analysis results of the Class-J PA at 5 GHz centre frequency, whose OMN is designed by considering Zout of the M1 transistor as the R_opt_ value, are obtained in the same manner as explained for the Class-J PA designed at 850 MHz centre frequency in Fig. 13 are shown below in Fig. 22.

In addition to the above parameters, another main figure of merit that evaluates the performance of PA is its linearity. In general, the linearity of the PA can be measured in various terms like P1dB (1 dB compression point), third-order intercept point, AM-PM distortion, Adjacent Channel Power Ratio (ACPR) etc. Initially, the RF input of the designed class -J PA in this work is a continuous wave (CW) signal. Therefore, the linearity of this PA is measured using only 1 dB compression point.

**Figure 23:**
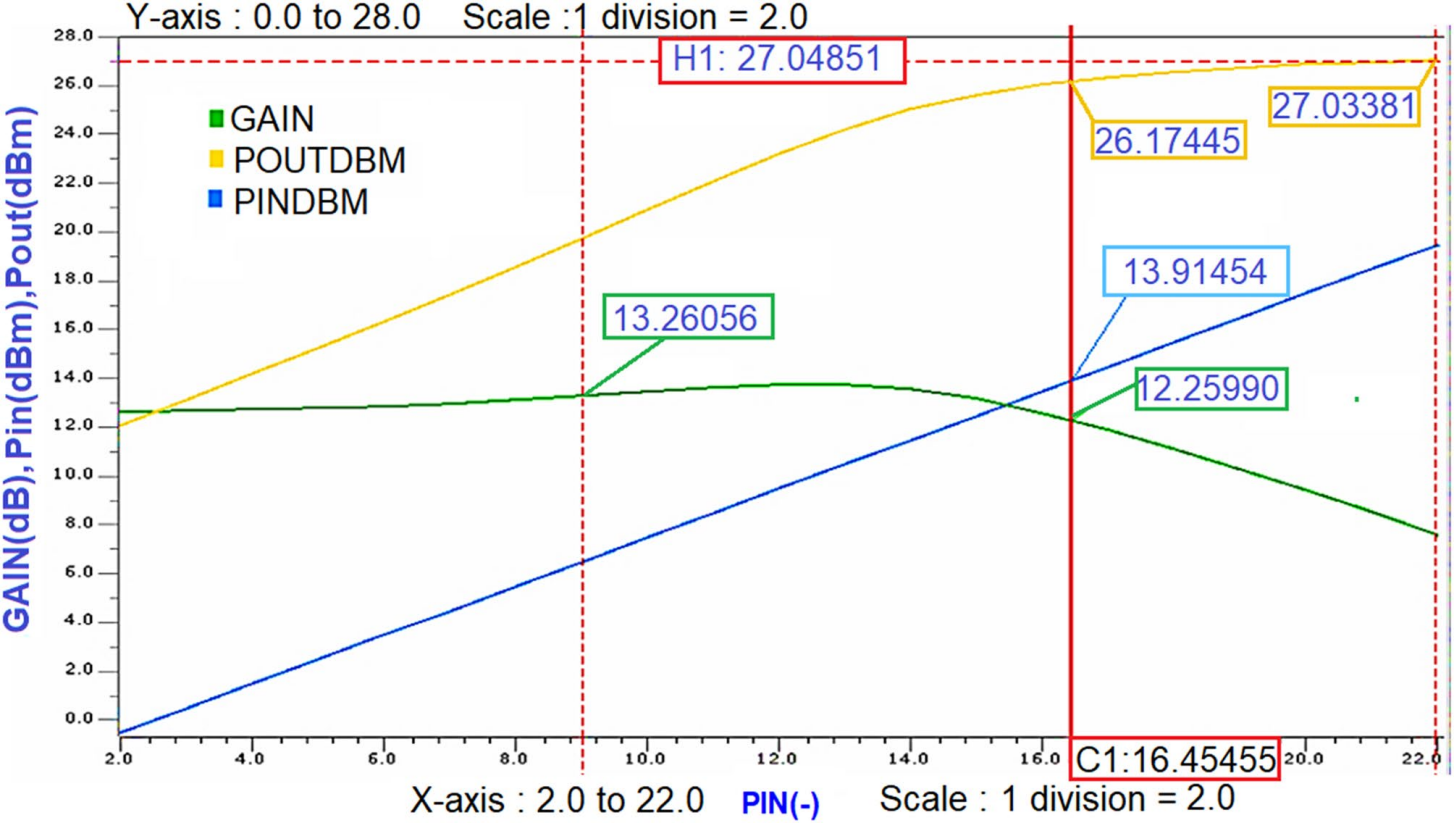
The 1 dB compression point and saturated power (Psat) of Class-J PA.

From the simulation results, it is noticed that the gain is compressed to 1 dB (i.e., from 13.3 dB to 12.3 dB), and the Pout corresponding to that 1 dB compression point is 26.2 dBm as shown in Fig. 23, which is almost near to the Psat value (i.e., 27 dBm) reveals that the designed Class-J PA is linear.

**Figure 24:**
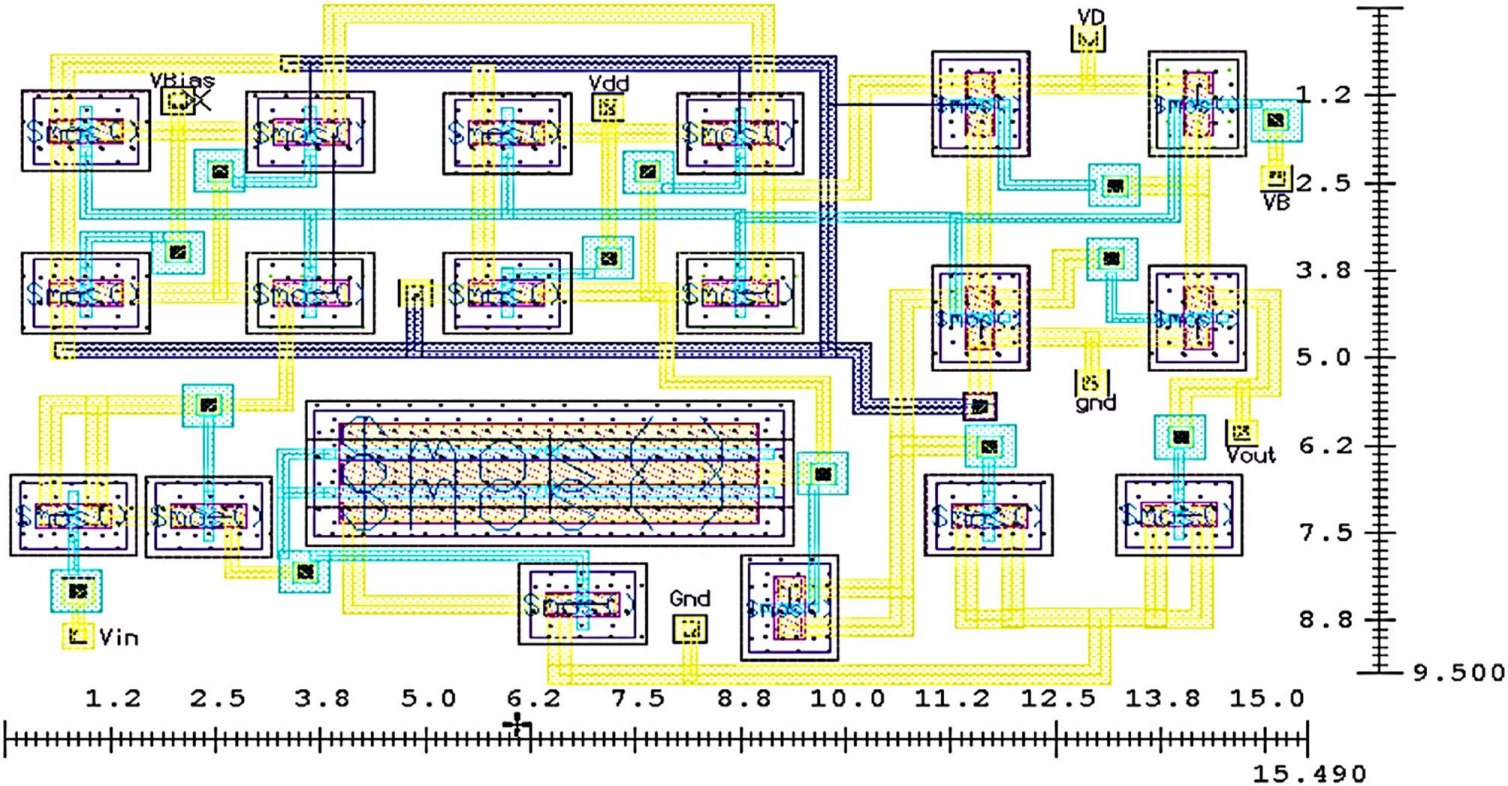
The core layout of Class-J PA (9.5 × 15.5) μm^2^.

Once the performance parameters of PA are validated from the simulation results, the schematic circuit’s layout design is initiated to estimate the chip size of the designed PA circuit. As the Class-J PA designed in this work is based on the passive lumped elements, all these elements of the schematic circuit are replaced with the active elements (i.e., transistors) such as an active inductor, capacitor, and resistor for layout design to reduce the chip area of the PA circuit. The layout design of the Class-J PA is performed using mentor graphics EDA tool. All the transistors are optimized and arranged as compact as possible to achieve a smaller chip area without compromising the performance of the PA. The layout design of Class-J PA with estimated chip size is shown in Fig. 24.

The performance parameters of Class-J PA designed at different frequencies were computed with .EXTRACT commands using the SST analysis command window are summarized in Table 1. It can be observed that the Class-J PA designed at RF input frequency of 850 MHz with a Q factor of 4 delivers 24 dBm of maximum power output with a maximum power gain of 23.8 dB. over a BW of 212 MHz for a 5 V supply to a 50 Ω load. However, when the same class J PA is designed at the RF input frequency of 5 GHz (i.e., sub 6 GHz Freq) with a Q factor of 10, it delivers a maximum power output of 26 dBm with a maximum power gain of 10.5 dB over the BW of 500 MHz. It is also observed that a maximum power output of 27 dBm with a maximum power gain of 13.7 dB. Over the BW of 500 MHz was obtained when the same Class-J mode PA was designed based on the Ropt value obtained, including Zout of transistor extracted from the S-parameter analysis at 5 GHz RF input frequency.

**Table 1:** Performance comparison of designed Class-J PA.

Value	Frequency 850 MHz	Frequency 5 GHz	Frequency 5 GHz
Feature	Class-J	Class-J	Class-J
Freq	850 MHz	5 GHz	5 GHz
VSupply [V]	5	5	5
Gain [dB]	23.8	10.5	13.7
Pout[dBm]	24	26	27
PAE max [%]	16	20	6.5
DE max [%]	18	13	8
BW [MHz]	212	500	500

The performance parameters of Class-J PA presented in this work are compared with the recent similar Class-J PA designs reported in the literature review, as shown in Table 2. However, it is difficult to compare their performances as they were designed at different frequencies and technologies our Class-J PA delivered a P_sat_ of 27 dBm without stacked structure compared to a 2-stacked Class-J PA presented in Ref.^15^ that delivers a Psat of 22dBm. A 28 nm CMOS Class-J PA designed at MM wave frequency of 28 GHz in Ref.^17^ delivers a Psat of 16.2dBm and power gain of 15 dB with 2 -stage structure, whereas the single-stage Class-J PA of this work can deliver a Psat of 27dBm with a power gain of approximately13.7 dB. The Class-J PA presented in Ref.^20^ designed with GaAs technology shows its linearity by delivering the OP1dB of 20 dBm that is almost nearer to the Psat of 21dBm similarly, the Class-JPA of this work delivers an OP1dB of 26.2 dBm that is almost nearer to the Psat of 27dBm to exhibits its linearity. A 130 nm CMOS-based 2-stage stacked Class-J PA delivers an OP1dB of 18 dBm, and a Psat of 20.2dBm with a max power gain of 15 dB is presented in Ref.^21^, whereas this work’s Class-J PA delivers Psat of 27 dBm with a maximum power gain of 13.7 dB with single-stage structure. However, the Class-J PA designed in this work exhibits good linearity and power output over desired BW. The other performance parameters of the PA need to be enhanced further to make it more appealing for emerging 5G applications.

**Table 2:** Performance comparison of recent Class-J PAs.

	2017^15^	2017^17^	2019^20^	2021^21^	This work
Technology	180 nm CMOS	28 nm CMOS	Avago GaAs	130 nm CMOS	130 nm CMOS
Feature	Class-J (stacked)	Class-J	Class-J	Class-J (stacked)	Class-J
No of stages	1	2	1	2	1
VSupply [V]	3.3	1	7	1.5 and 2.5	5
Frequency. [GHz]	3.5	28	2.4	9.5	5
BW [GHz]	2.1–4.5	22–34	–	8.7–11.8	4.3–5.4
OP1dB[dBm]	**–**	**–**	20	18	26.2
PSat[dBm]	22	16.2	21	20	27
Max power gain [dB]	17.4	15	–	15	13.7

## Conclusion

A 5 GHz Class-J PA with output matching designed using a lumped π-type network is presented in this paper. This Class-J PA obtained a peak output power of 27 dBm, a maximum power gain of 13.7 dB, and a small signal gain of 17 dB at 5 GHz with a BW of approximately 500MGHz.A small overlap is observed between the half-wave rectified voltage and current waveforms at the drain, which is the Class-J mode PA's key feature. Although this waveform shows the feature of switching mode PA (i.e., the power dissipation across the transistor is very less), this PA’s OP1Db reveals that it can provide the linearity similar to the class-B or AB modes due to its non-switching mode of operation. There is no need for harmonic traps, unlike in class B, which makes the Class-J PA topology simple and more appealing for broadband applications. This Class-J PA's active element-based-layout design indicates the feasibility of the designed PA circuit for chip integration. However, this work is in progress to enhance the other performance parameters such as PAE, DE, and BW.
